# Emergency inguinal hernioplasties in a tertiary public Hospital in Athens Greece, during the economic crisis

**DOI:** 10.1186/s12893-019-0477-9

**Published:** 2019-02-04

**Authors:** Ioannis G. Karavokyros, George I. Kirkilessis, Demetrios Schizas, Georgios Chelidonis, Emmanouil Pikoulis, John Griniatsos

**Affiliations:** 1 1st Department of Surgery, Medical School, National and Kapodistrian University of Athens, Laikon General Hospital, Agiou Thoma 17str, 115-27 Athens, GR Greece; 2 National Actuarial Authority, 29 Stadiou Str, 101-10 Athens, GR Greece

**Keywords:** Inguinal hernia, Hernioplasty, Economic crisis, Greece

## Abstract

**Background:**

Although the effect of the recent Greek economic crisis and austerity on the population’s health and the health system effectiveness have been discussed a lot recently, data on common surgical conditions affecting large part of the population are missing. Using inguinal hernia as a model we investigated possible changes of citizens’ attitude regarding the time of referral, the perioperative details and the intraoperative findings of the emergency hernioplasties.

**Methods:**

The present retrospective study was conducted by a Department of Surgery in a tertiary public hospital of the Greek capital. We reviewed the records of all hernioplasties performed during two 5-year periods: 2005–2009 and 2012–2016, i.e. before and during the crisis focusing on the emergency ones (either incarcerated or strangulated).

**Results:**

An equal number of hernioplasties was performed in both periods. During the crisis however, an emergency hernioplasty was significantly more probable (HR 1.269, 95% CI 1.108–1.1454, *p = 0.001*), at a younger age (*p = 0.04*), mainly in patients younger than 75 years old (*p = 0.0013*). More patients presented with intestinal ischemia (7 vs 18, *p = 0.002*), requiring longer hospitalization (5.2 vs 9.6 days*, p = 0.04*), with higher cost (560 ± 262.4€ vs 2125 ± 1180.8€ *p* < 0.001). In contrast the percentage of patients with intestinal resection, their hospitalization length and treatment-cost remained unchanged. During the crisis there was a non-significant increase of emergency patients requiring ICU postoperatively (0 vs 4, *p = 0.07*) and a non-significant 60% increase of emergency operations in migrants/refugees population (3.5% vs 5.8%, *p = 0.28*). Epidural anesthesia was significantly more frequent during the crisis.

**Conclusion:**

During the crisis: (i) the emergency hernioplasties increased significantly, (ii) more patients (exclusively Greek) presented with intestinal ischemia requiring longer hospitalization and higher treatment cost, (iii) the mean age of the urgently treated patients decreased significantly (iv) regional (epidural) anesthesia was more frequent. Although a direct causal relation could not be proven by the present study most observations can be explained by an increase of the patients who delayed the elective treatment of their hernia, and by a redistribution of the surgical workload towards big central hospitals. This can be prevented by adequately supporting the small district hospitals.

## Background

Economic recession has affected many European countries since 2008. Its effect on both the health of the European citizens, and on the quality of the provided services by the healthcare systems has attracted much attention. For Greece in particular, the protracted and severe financial crisis was combined with strict austerity measures implemented by the Greek Government since the beginning of 2010. Well documented effects of this combination are the increase of the burden of COPD [1], and its pharmacological treatment cost [2], increased death rates from nervous system diseases and mental health problems [3], increased deaths due to adverse events during medical treatment [4], increased rates of suicide attempts particularly in males [5], decrease by nearly 50% of the number of solid organ transplantations from deceased donors [6], poor out-of-hospital outcome of acute coronary syndrome in patients with low annual income [7], decrease of both the duration and the completion of anti-tuberculosis therapy [8], increased infant mortality [9] and dramatic decrease (by 15%) in livebirths rate [10]. In total, the health indicators for the Greek population worsened more than those of other countries similarly affected by recession [11]. Crisis has been appreciated as detrimental for the population health and the Healthcare Systems [12] but on the same time, as opportunity for reform and improvement [13]. In this aspect a number of methods have been proposed for reduction of the cost of the daily surgical practice [14–17]. However, to the best of our knowledge, no report from Greece has been published yet on how the Greek citizens deal with a surgical problem during the crisis era. On this purpose, we studied inguinal hernia and its treatment by inguinal hernioplasty, a common surgical condition and its cure by a routine operation. We particularly focused on incarcerated or strangulated hernias which by definition call for an emergency, and thus unscheduled, operation.

## Methods

### Patients

This study was conducted by a University Department of Surgery settled at “Laiko Hospital”, a public tertiary general hospital in the center of Athens. The hospital is subjective to the rules of the Greek National Health System (GNHS) and serves as one out of the four chief Central Hospitals for the Greek capital in the GNHS on-call-rota. According to the GNHS rules, everybody can visit the Emergency Department of any on-call hospital (district or central) whenever he feels like to do so. Every eighth day, our Department covers the surgical emergencies for most of the capital’s population. Consequently, our practice reflects the surgical needs of the inhabitants of the Greek capital and the ability of the GNHS to provide service to its citizens. In both study periods the service provided by the GNHS was free of charge for those patients having a valid Social Security Status. With very few exceptions (as for example those listed in the Joint Ministerial Decision 139491/2006) the rest of the patients were charged with the cost of their treatment. In 2014 a change towards free service to those affected by the crisis was attempted, but it was no sooner than 2016 when free treatment was granted to almost everybody (including those unable to maintain a valid Social Security Status) after the implementation of the Law 4368/2016 and the Joint Ministerial Decision 25132/2016. We can therefore conclude that the GNHS service was not free for everybody and a percentage of our fellow citizens had to pay for their treatment in both periods examined.

We studied two 5-year periods, immediately prior to (2005–2009) and during the economic crisis (2012–2016). The years 2010 and 2011 were thought off as transitional and exempted from the study. In both periods the referral and the On-Call system of the GNHS were practically unaltered. We retrospectively reviewed the records of all the inguinal hernioplasties performed during these periods by the same group of surgeons. Patient’s age, elective or urgent (typically performed “out of hours”) nature of the operations, type of anesthesia were retrospectively analyzed. In particular we focused on emergency operations (i.e. in strangulated or incarcerated hernias) where we analyzed data regarding surgical technique, signs of ischemia of the herniated organ, concomitant intestinal resections, length of hospital stay (LOHS) and cost of treatment per patient.

### Operative technique

Since the same group of surgeons covered both study periods operative technique remained unaltered in time. In brief, after induction to the general endotracheal or epidural anesthesia (the choice of anesthesia was left on the anesthesiologist’s preference), a 6-10 cm inguinal incision was made on the affected site. In all emergency cases an open approach was chosen and always the hernia sac was incised and the protruded hollow viscus was evaluated. When signs of ischemia were detected, restoration of normal blood supply and viability of the protruded viscus were attempted by all means (eg. enlargement of the hernia ring, warm wet swabs, use of hypertonic solutions, etc) for 15 to 45 min. A viable intestinal loop was accommodated back to the abdominal cavity. Absence of viability signs in the affected intestinal loop after 45 min led to small bowel resection and construction of enteroanastomosis (end-to-end or side-to-side, hand sewed or stapled). All cases without small bowel resection were repaired with a tension-free method with mesh. All cases with intestinal resection were repaired without employing prosthetic mesh.

The presence of intestinal ischemia was confirmed through the pathology reports.

The cost of treatment per patient was available from the Accounting Department of the hospital.

In accordance to the Declaration of Helsinki, the present study was approved by the Scientific Council of the “Laiko” Hospital.

### Statistical analysis

Statistical comparisons were made with IBM SPSS® statistical package using t-test, Chi-square with Yate’s correction, and Fisher’s Chi-square. A *p* value < 0.05 was considered as statistically significant.

## Results

Table 1 lists patient demographics and the operative characteristics of all the hernioplasties performed before and during the crisis**.** In both periods we performed a similar number of operations. However, in the crisis period there were 53 less elective operations (decrease by 24%) and 46 more emergency ones (increase by 23%) leading to a statistically significant increase of the incidence of emergency inguinal hernioplasties (HR 1.269, 95% CI 1.108–1.1454, *p = 0.001*). The nationality and the sex distribution of patients among the two periods remained constant but epidural anesthesia was more frequent in the crisis era (27.5% vs 38.1%, *p* = 0.001).

**Table 1:** Demographics and Operative Characteristics of all inguinal hernioplasties performed before and during the economic crisis

Period/Parameter	2005–2009 (*n* = 414)	2012–2016 (*n* = 407)	*p*-value
Gender			
Male	401 (96.8%)	389 (95.6%)	
Female	13 (3.2%)	18 (4.4%)	*NS*
Nationality			
Greek Nationality	402 (97.1%)	386 (95%)	
Other Nationality	12 (2.9%)	21 (5%)	*NS*
Type of anaesthesia			
General endotracheal	300 (72.5%)	252 (61.9%)	
Epidural	114 (27.5%)	155 (38.1%)	*0.001*
Type of operation			
Elective hernioplasties	219 (52.89%)	166 (40.8%)	
Emergency hernioplasties	195 (47.1%)	241 (59.2%)	*0.001*

Table 2 summarizes patient demographics, operative and hospitalization features and treatment cost for the emergency operations conducted before and during the crisis**.** Analyzing this subgroup further we should mention that although the sex and nationality distribution remained unaltered during the crisis, the mean age of the patients was younger (67-years old vs 71-years old, *p = 0.04*). During the crisis period, less patients older than 75 years were operated urgently (48.2% vs 36.5%, *p* = 0.013). The crisis period was also characterized by a statistically significant increase of the patients with intraoperatively detected intestinal ischemia (3.6% vs 7.5% of the emergency operations, *p = 0.002*).These patients had a longer hospital stay (*p = 0.04*) and an increased average treatment-cost per patient (*p < 0.001*). On the other hand, the percentage of patients needing intestinal resection, their hospital stay and their average treatment-cost remained constant among the two periods (*p = 0.8, p = 0.2* and *p = 0.2* respectively). All three patients who had intestinal resection in the former period survived. In contrast one out of the seven patients of the crisis period passed away in the ICU due to his comorbidities. Finally, during the second period, more epidural anesthesias were used (42.6% vs 45.2%). Other non-significant changes seen during the crisis in the emergency setting was a 60% increase of migrants/refugees (3.6% vs 5.8%) and an increased need for postoperative ICU stay (0 out of 195 vs 4 out of 241).

**Table 2:** Demographics, Operative and Hospitalization Features, and Treatment Cost of the Emergency Hernioplasties before and during the economic crisis

Period/Parameter	2005–2009 (*n* = 195)		2012–2016 (*n* = 241)		*p-value*
Gender					
Male	188 (96.4%)		227 (94.2%)		
Female	7 (3.6%)		14 (5.8%)		*NS*
Age (years)					
(Mean ± SD)	71,1 ± 15,1		66,7 ± 14,7		*0.04*
Age (Distribution by decade)					
< 35	0	0%	5	2.1%	
35–44	9	4.6%	9	3.7%	
45–54	27	13.8%	39	16.2%	
55–64	29	14.9%	38	15.8%	
65–74	36	18.5%	62	11.5%	
75–84	51	26.2%	57	23.7%	
≥85	43	22.1%	31	12.7%	*NS*
Age					
< 75 years	101 (51.8%)		153 (63.5%)		
≥ 75 years	94 (48.2%)		88 (36.5%)		0.013
Nationality					
Greek	188 (96.4%)		227 (94.2%)		
Other	7 (3.5%)		14 (5.8%)		*NS*
Type of anaesthesia					
General endotracheal	112 (57.4%)		132 (54.8%)		
Epidural	83 (42.6%)		109 (45.2%)		
Cases with bowel ischemia (% of emergency operations)	7 (3.6%)		18 (7.5%)		*0.002*
Intestinal resection (% of cases with ischemia)	3 (43%)		7 (39%)		*NS*
Mortality in cases of intestinal resection	0		1		*NA*
No of pts. who needed ICU	0		4 (1.7%)		*NA*
LOHS^a^ in cases with ischemia (days) (Mean)	5.2		9.6		*0.04*
LOHS in cases of intestinal resection (days) (Mean)	7		11.2		*NS*
Average cost in cases with ischemia (€) (Mean ± SD)	560 ± 262.4		2125 ± 1180.8		*< 0.001*
Average cost in cases with resection (€) (Mean ± SD)	848 ± 199.6		2536 ± 1339.4		*NS*

^a^*LOHS* length of hospital stay, *SD* standard deviation

## Discussion

Although the global debt crisis started from USA in 2008, the Greek Government signed the first Economic Adjustment Program in May 2010. In the beginning of 2011 significant austerity measures were implemented and their consequences became obvious from the beginning of 2012. Thus, the true economic crisis, affecting the vast majority of the Greek citizens, started in 2012 and it seems that it continues uninterruptedly till now.

In the present small retrospective study we compared two representative periods of time: prior to (2005–2009) and during the economic crisis (2012–2016), using as a model the natural course of the inguinal hernia. The primary end-point of the study was the evaluation of the citizens’ attitude regarding the time of presentation to the hospital for inguinal hernia repair. Secondary end-points were the perioperative details and intraoperative findings of the emergency hernioplasties. What we found is that although an equal number of inguinal hernioplasties were performed in both periods, the later one was characterized by a decrease of elective and an increase of emergency operations, an increase of the patients who presented with intestinal ischemia, a prolongation of their hospital stay and a higher cost of their treatment, as well as an increased number of patients needing ICU hospitalization although this did not reach statistical significance. Interestingly these parameters remained unaffected by the crisis for the subgroup of patients requiring bowel resection.

It is well known that an urgent operation due to incarceration or strangulation will be needed in 5–15% of those inguinal hernias who are left untreated [18, 19]. Furthermore approximately 15% of these emergency operations will lead to intestinal resection due to irreversible bowel ischemia [20]. The risk of resection increases with advanced age and delayed presentation [20].

Prior to the economic crisis, the Greek healthcare system was among the most “privatized” between EU countries [21], having one of the largest shares of public health expenditure given that it constituted the 39.7% of the total health expenditure [22]. Elective inguinal hernioplasties were mainly performed in private and district public hospitals, leaving only selected or emergency cases for treatment in central, tertiary or teaching ones.

Evidence from past economic crises implies that the decreased household income moves the consumption of health services from the private sector towards the public one [23]. In our case this would translate to an increase of our elective hernioplasties or, in case we had reached our capability limits, by a stability of elective hernioplasties together with an elongation of our waiting list. To our surprise the number of elective operations neither increased nor remained stable in the crisis period. In contrast they dropped significantly followed by a disproportionate increase of the emergency operations, given that only 5–15% of the untreated hernias proceed to strangulation [18, 19]. In addition, the incidence of intestinal ischemia in the crisis era increased even more disproportionate by more than 100%. These findings altogether (i.e. decrease of the elective, disproportionate increase of the emergency, and even more disproportionate increase of the cases with ischemia) can be offered by a possible increase of the patients who, during the crisis and for whatever reason, left their hernias untreated and postponed its elective repair. A percentage of these untreated hernias eventually developed complications, forcing the patients urgently to a public on-call hospital. As literature cites this percentage should have been around 15% [18, 19] but we evidenced a much higher increase, as if our catching population enlarged during the crisis. It highly probable that under the influence of dramatic media reports [24–26] for lack of personnel, lack of materials and dismantling infrastructures in the small public hospitals, some patients bypassed the district hospitals asking for help from the tertiary ones, like ours. This could explain all the changes we evidenced: the decrease of the elective and the disproportionate increase of the emergency workload, as well as the tremendous increase of the cases with ischemia. This lack of proportion could rest in that we received patients who, in other times, would have addressed the private or the small district hospitals. The present study cannot verify the validity of our scenario. On the other hand, it is understandable that if the small district hospitals were adequately supported, then the media would have been unable to affect population psychology in their attempt to attract public attention, and that the patients would have been distributed across the GNHS hospitals in a more reasonable way.

Greece faces a migrant and refugee crisis also [27] and these populations experience severe barriers in access to healthcare system [28], while they are characterized by increased hospitalization rates in cases of acute surgical or accident-related diagnoses [29]. Thus, somebody could postulate that the migrants/refugees population could be responsible for our results. Although we noticed a 60% increase (7 out 195 vs 14 out of 241) of emergency operations on migrants/refugees during the crisis, they still represented less than 6% of the total number of the emergency operations, a small percentage to explain our findings. Furthermore none of the refugees presented with intestinal ischemia and none of them stayed in the ICU postoperatively. Intestinal ischemia and intestinal resections exclusively affected Greek citizens.

Leaving a known hernia temporarily unrepaired, as we suggested that some of our fellow citizens did, could be due to their inability to afford the cost of a private hospital and the compulsory enrollment in the waiting list of a public district hospital. Alternatively it could simply be due to negligence. A long standing and asymptomatic inguinal hernia can be erroneous appreciated as a minor problem. Neglecting a timely repair and adopting a “see how it goes” attitude is in accordance with what has already been described for other medical conditions [30, 31]. Non-utilization of healthcare services by those citizens in need of care has been correlated with the reduction of income due to unemployment; with the fear for a possible loss of income or even job loss after sick-leave; and with the drastic cuts in salaries and pensions [32]. In such a case some patients would seek treatment only when their hernia would become symptomatic, but at that time, the probably pre-existing and equally uncontrolled comorbidities would potentially demand ICU hospitalization even for an inguinal hernioplasty.

The present study disclosed that the patients who were operated as an emergency during the crisis were significantly younger than the ones prior to the crisis. This is partially in agreement to historic documentations [33, 34] that populations less than 65 years old are those affected harder by economic crises (unemployment, lack of insurance, job insecurity, loss of the disposable income, increased morbidity); and that only in cases of emergency they resort to public health care systems. Implementation of austerity measures in Greece affected equally pensions and salaries and prohibited old and younger patients equally from an elective hernia repair. Perhaps this contributed to the higher age of the affected population we observed.

Another finding was the small but statistically significant change towards regional anesthesia for inguinal hernioplasty in both elective and emergency setting during the second period. We have no data to elucidate the reasons of this change and we have no explanation for the observed high percentage of general anesthesia in both periods. Perhaps it was the natural evolution of the daily anesthesiologists’ practice from old habits to modern science. The advantage and cost effectiveness of regional anesthesia compared to the general one for operations of short duration in high risk patients are well documented [35, 36]. On the other hand, perhaps this change may relate to the reduced public health expenditures (including the hospital budgets) by 5.5 billion euros between 2009 and 2012 [37], a fact not leaving unaffected the financing of any public hospital’s Unit or Department. In any case, this observation cannot be interpreted in the frames of the present study and we simply state it as a point worthy of further investigation.

The last finding of the present study was that the hospital cost in cases with ischemia quadrupled during the crisis. However this should be appraised critically. Before 2011, the calculation of the daily hospital cost for the in-patients was based on unrealistic low hospital-charges which remained unchanged since the beginning of 1990s. In 2012, a Diagnosis Related Grouping system (“KEN” / DRGs) was implemented. This was based on the ICD-10 coding system for diseases and aimed to rationalize the actual cost of hospitalization and its reimbursement. Since the 18 most high-volume DRGs in Greek hospitals were comparable to their Australian and German counterparts in terms of price, excluding personnel payroll [38], we assume that the new hospital-charges are more realistic than the past ones. The authors of the present study are not suitable for econometric analyses and as result cannot conclude whether current hospital cost is “truly high” or the past one was “falsely low”. Moreover whether the newly implemented DRGs system improved the hospitals economic self-reliance, remains out of the scope and the ability of the present study.

This is a small retrospective study performed in one department of a single tertiary hospital in the capital of Greece on a topic where little knowledge exists. It is therefore inherently limited by its retrospective nature, by its size and by the lack of existing evidence. The influence of unknown confounding factors cannot be ruled out. Furthermore Greek legislation obliges hospitals to maintain records and data kept in paper for a period of 10 years. Interesting variables for the first period, needed for a detailed cost analysis (e.g. price of the theater dispensables) are no longer available and we used only data retrieved from the hospital computers. Similarly re-admission rates were not taken under consideration given that patients had the legal right and could have been readmitted in other hospitals. Nevertheless, and despite its shortcomings, our study has suggested some associations, although a solid causative relation was not proven. This can only derive from properly designed, larger prospective studies enrolling more Departments and more hospitals of both private and public sector. As economic crisis is an unexpected and unwanted event, designing and conducting prospective studies on population health and population attitude in such situations will remain a difficult, if not impossible, task.

## Conclusions

To the best of our knowledge, the present study is the first one coming from a tertiary central public hospital in Greece, evaluating if and how the recent economic crisis and austerity measures affected the attitude of the Greek citizens facing a surgical problem, as well as their effect on the healthcare system services.

Using as a model the natural course of the inguinal hernia, in two distinct 5-year periods of time (ie. before and after the crisis), the present study concluded that during the later period: (i) the emergency hernioplasties have increased significantly, (ii) exclusively Greek patients presented with intraoperative findings of intestinal ischemia requiring longer hospitalization and higher treatment cost, while the percentage of patients with intestinal resections, their hospitalization and treatment cost remained unchanged, (iii) the mean age of the urgently treated patients has decreased significantly (iv) the need for postoperative ICU stay increased (v) admissions and emergency operations rate for migrants and refugees also increased (vi) regional (epidural) anesthesia was more frequently employed. Most observations can be explained by an increase of the patients who, for whatever reason, either postponed or neglected the elective treatment of their hernia and by a redistribution of the surgical workload towards big central hospitals. Perhaps adequate support to small district hospitals would alleviate this phenomenon and assist in keeping the surgical workload distributed more rationally across the GNHS hospitals.

## Abbreviations

“KEN” / DRGs: Diagnosis Related Groups
GNHS: Greek National Health System
ICU: Intensive Care Unit
LOHS: Length Of Hospital Stay

## References

[1] Kotsiou OS, Zouridis S, Kosmopoulos M, Gourgoulianis KI. Impact of the financial crisis on COPD burden: Greece as a case study. Eur Respir Rev 2018;27:170106. doi: 10.1183/16000617.0106-2017. 10.1183/16000617.0106-2017 PMC9489147 29367410

[2] Stafyla E, Kerenidi T, Gerogianni I, Geitona M, Daniil Z, Gourgoulianis KI (2017). The pharmacological cost of COPD during Greek economic crisis. Int J Chron Obstruct Pulmon Dis.

[3] Laliotis I, Ioannidis JPA, Stavropoulou C (2016). Total and cause-specific mortality before and after the onset of the Greek economic crisis: an interrupted time-series analysis. Lancet Public Health.

[4] McKee M, Stuckler D (2016). Health effects of the financial crisis: lessons from Greece. Lancet Public Health.

[5] Papaslanis T, Kontaxakis V, Christodoulou C, Konstantakopoulos G, Kontaxaki MI, Papadimitriou GN (2016). Suicide in Greece 1992-2012: a time-series analysis. Int J Soc Psychiatry.

[6] Moris D, Zavos G, Menoudakou G, Karampinis A, Boletis J (2016). Organ donation during the financial crisis in Greece. Lancet.

[7] Andrikopoulos G, Tzeis S, Terentes-Printzios D, Varounis C, Vlachopoulos C, Mantas I (2016). Impact of income status on prognosis of acute coronary syndrome patients during Greek financial crisis. Clin Res Cardiol.

[8] Sotiropoulou P, Gourgoulianis K, Konstantinou K, Petinaki E, Roupa Z (2015). Retrospective study of measuring tuberculosis therapy compliance: Greece as a host country for vulnerable populations before and during the financial crisis. Mater Sociomed.

[9] Rajmil L, Fernandez de Sanmamed MJ, Choonara I, Faresjö T, Hjern A, Kozyrskyj AL (2014). Impact of the 2008 economic and financial crisis on child health: a systematic review. Int J Environ Res Public Health.

[10] Vrachnis N, Vlachadis N, Iliodromiti Z, Vlachadi M, Creatsas G (2014). Greece's birth rates and the economic crisis. Lancet.

[11] Bacigalupe A, Shahidi FV, Muntaner C (2016). Why is there so much controversy regarding the population health impact of the great recession? Reflections on three case studies. Int J Health Serv.

[12] Kentikelenis A, Karanikolos M, Papanicolas I (2011). Health effects of financial crisis: omens of a Greek tragedy. Lancet.

[13] Liaropoulos L (2012). Greek economic crisis: not a tragedy for health. BMJ.

[14] Sotiropoulos GC, Spartalis E, Machairas N, Kouraklis G. Peripheral hepatojejunostomy: a last resort palliative solution in Greece during the economic crisis. BMJ Case Rep. 2017. 10.1136/bcr-2016-217368. 10.1136/bcr-2016-217368 PMC5612007 28765486

[15] Arkadopoulos N, Gemenetzis G, Danias N, Kokoropoulos P, Koukopoulou I, Bartsokas C (2016). Cost-effective surgical management of liver disease amidst a financial crisis. World J Surg.

[16] Stavrou G, Fotiadis K, Panagiotou D, Faitatzidou A, Kotzampassi K (2015). Homemade specimen retrieval bag for laparoscopic cholecystectomy: A solution in the time of fiscal crisis. Asian J Endosc Surg.

[17] Manatakis DK, Georgopoulos N. Reducing the cost of laparoscopy: Reusable versus disposable laparoscopic instruments. Minim Invasive Surg. 2014;Article ID 408171. 10.1155/2014/408171. 10.1155/2014/408171 PMC4134811 25152814

[18] Andrews NJ (1981). Presentation and outcome of strangulated external hernia in a district general hospital. Br J Surg.

[19] Kulah B, Kulacoglu IH, Oruc MT (2001). Presentation and outcome of incarcerated external hernias in adults. Am J Surg.

[20] Kurt N, Oncel M, Ozkan Z (2003). Risk and outcome of bowel resection in patients with incarcerated groin hernias: retrospective study. World J Surg.

[21] Grigorakis N, Floros C, Tsangari H, Tsoukatos E (2016). Out of pocket payments and social health insurance for private hospital care: evidence from Greece. Health Policy..

[22] Economou C (2010). Greece: Health system review. Health Syst Transit.

[23] Lloyd-Sherlock P (2005). Health sector reform in Argentina: a cautionary tale. Soc Sci Med.

[24] Smith H. Patients who should live are dying: Greece’s Public Health meltdown. Guardian. 2017. Available from https://www.theguardian.com/world/2017/jan/01/patients-dying-greece-public-health-meltdown. Accessed 28 Jan 2019.

[25] De Filippis V. Grèce : le «malade imaginaire» perdu dans un labyrinth. Liberation. 2017. Available from http://www.liberation.fr/debats/2017/02/27/grece-le-malade-imaginaire-perdu-dans-un-labyrinthe_1551425. Accessed 28 Jan 2019.

[26] Spiliopoulou M, Anagnostopoulou V. Austerity-hit Greek Public Health system struggling with shortages, PM pledges improvement. Xinhua News Agency. Available from http://news.xinhuanet.com/english/2017-03/19/c_136140632.htm Accessed 28 Jan 2019.

[27] Tsiamis C, Terzidis A, Kakalou E, Riza E, Rosenberg T (2016). Is it time for a Refugees’ Health Unit in Greece?. Lancet.

[28] van Loenen T, van den Muijsenbergh M, Hofmeester M, Dowrick C, van Ginneken N, Mechili EA (2018). Primary care for refugees and newly arrived migrants in Europe: a qualitative study on health needs, barriers and wishes. Eur J Pub Health.

[29] Tsitsakis CA, Karasavvoglou A, Tsaridis E, Ramantani G, Florou G, Polychronidou P (2017). Features of public healthcare services provided to migrant patients in the eastern Macedonia and Thrace region (Greece). Health Policy..

[30] Zavras D, Zavras AI, Kyriopoulos II (2016). Kyriopoulos J. Economic crisis, austerity and unmet healthcare needs: the case of Greece. BMC Health Serv Res.

[31] Pappa E, Kontodimopoulos N, Papadopoulos A, Tountas Y, Niakas D (2013). Investigating unmet health needs in primary health care services in a representative sample of the Greek population. Int J Environ Res Public Health.

[32] Karanikolos M, Mladovsky P, Cylus J, Thompson S, Basu S, Stuckler D (2013). Financial crisis, austerity, and health in Europe. Lancet.

[33] Kondilis E, Giannakopoulos S, Gavana M, Ierodiakonou I, Waitzkin H, Benos A (2013). Economic crisis, restrictive policies, and the population’s health and health care: the Greek case. Am J Public Health.

[34] Mechili AE, Kalokairinou A, Kaitelidou D, Diomidous M (2015). Greece financial crisis and quality of life. Stud Health Technol Inform.

[35] Finsterwald M, Muster M, Farshad M, Saporito A, Brada M, Aguirre JA (2018). Spinal versus general anesthesia for lumbar spine surgery in high risk patients: perioperative hemodynamic stability, complications and costs. J Clin Anesth.

[36] Siu A, Patel J, Prentice HA, Cappuzzo JM, Hashemi H, Mukherjee DA (2017). Cost analysis of regional versus general anesthesia for carotid endarterectomy. Ann Vasc Surg.

[37] Souliotis K, Golna C, Tountas Y, Siskou O, Kaitelidou D, Liaropoulos L (2016). Informal payments in the Greek health sector amid the financial crisis: old habits die last. Eur J Health Econ.

[38] Polyzos N, Karanikas H, Thireos E, Kastanioti C, Kontodimopoulos N (2013). Reforming reimbursement of public hospitals in Greece during the economic crisis: implementation of a DRG system. Health Policy.

